# Contribution of immunoglobulin products in influencing seasonal influenza infection and severity in antibody immune deficiency patients receiving immunoglobulin replacement therapy

**DOI:** 10.3389/fimmu.2024.1452106

**Published:** 2024-10-22

**Authors:** Mark Ballow, Raúl Ortiz-de-Lejarazu, Isabella Quinti, Matthew S. Miller, Klaus Warnatz

**Affiliations:** ^1^ Division of Allergy & Immunology, Morsani College of Medicine, University of South Florida, at Johns Hopkins All Children’s Hospital, St. Petersburg Florida, FL, United States; ^2^ Professor of Microbiology, Scientific Advisor & Emeritus Director, National Influenza Center, Valladolid, Spain; ^3^ Department of Molecular Medicine, Sapienza University of Rome, Head of the Primary Immunodeficiency Unit, Rome, Italy; ^4^ Michael G. DeGroote Institute for Infectious Disease Research, Department of Biochemistry & Biomedical Sciences, McMaster University, Hamilton, ON, Canada; ^5^ Department of Rheumatology and Clinical Immunology, Medical Center – University of Freiburg, Freiburg, Germany; ^6^ Center for Chronic Immunodeficiency (CCI), Medical Center – University of Freiburg, Freiburg, Germany; ^7^ Department of Clinical Immunology, University Hospital Zurich, Zurich, Switzerland

**Keywords:** immunoglobulin replacement therapy, influenza, primary immunodeficiency, secondary immunodeficiency, vaccination

## Abstract

Seasonal and pandemic influenza infection present a potential threat to patients with antibody deficiency. The acceptance and effect of the current recommendation for annual vaccination against influenza for patients with antibody deficiency is not well investigated and due to antigenic drift or shift the protective capacity of regular IgG replacement therapy (IgRT) is considered low. This narrative review considers the effect of influenza vaccination in immunodeficient patients and discusses available information on the effect of immunoglobulin products on seasonal influenza infectivity and severity in antibody deficiency patients receiving IgRT. The humoral immune response to seasonal influenza vaccination is reduced in patients with antibody immune deficiency. However, there is no evidence that the proportion of patients with primary antibody deficiency who develop influenza illness, and the severity of such illness, is increased when compared with the general population. The IgRT that patients receive has been shown to contain neutralizing antibodies as a consequence of past flu infections against both the hemagglutinin and neuraminidase surface proteins and other viral internal proteins of different influenza A virus strains. Studies have demonstrated not only significant levels of specific but also cross-reactive antibodies against seasonal influenza virus strains. Thus, despite the yearly changes in influenza viral antigenicity that occur, IgRT could potentially contribute to the protection of patients against seasonal influenza. Currently, only limited clinical data are available confirming a preventative effect of IgRT with respect to seasonal influenza infection. In conclusion, there is some evidence that IgRT could contribute to protection against seasonal influenza in patients with antibody-related immunodeficiency. However, additional clinical data are needed to confirm the extent and relevance of this protection and identify the main responsible virus targets of that protection.

## Introduction

Influenza is a highly contagious viral respiratory infection that causes pandemics and seasonal epidemics. Approximately 2 billion cases of seasonal influenza occur each year, including 3–5 million severe cases (1), and it is estimated to cause 389,000 (uncertainty range 294,000–518,000) respiratory-related deaths annually (2). Certain groups are at high risk of severe influenza infection, including children aged 6 months to 5 years, adults aged >65 years, pregnant women, and people with chronic medical conditions, including those who are immunocompromised (1, 3, 4).

The prevalence of influenza decreased during the first waves of the COVID-19 pandemic, due to non-pharmaceutical interventions (NPI) such as lockdown, quarantine, mass gathering limitations and the use of face-masks. More widespread circulation of influenza occurred from late 2021 as these interventions were reduced or discontinued (5). There is evidence that the severity of influenza has increased among children in the wake of the COVID-19 pandemic, possibly due to reduced population-level immunity in young children who are at elevated risk of more serious infection (6).

Vaccination is the primary method of preventing seasonal influenza infection (7, 8). It is especially important for those people who are at high risk of influenza infection and associated complications mentioned above, as well as their families and caregivers, and healthcare workers (1). Currently, there is a lack of uniform policy for seasonal influenza vaccination in the immunocompromised patient population, which includes those receiving immunosuppressive therapy, those with other forms of secondary immunodeficiency (SID, for example, due to hematological malignancy), and those with primary immunodeficiencies (PID or inborn errors of immunity) (9).

The most common type of PID is primary antibody deficiency (PAD), which accounts for approximately half of such disorders (10). Immunization is a key strategy for the prevention of seasonal influenza in patients with PID (10–12). However, little information is available about how many patients with PID receive the seasonal influenza vaccination, or how many become ill with influenza during the season. In addition, patients with antibody immune deficiency generally receive immunoglobulin replacement therapy (IgRT), which contains a range of antibodies from donor plasma that provide passive immunity against a range of pathogens to the patient (10). However, the effect of IgRT on the frequency of seasonal influenza diagnosis and severity of symptoms is not clear.

This narrative review summarizes the effect of influenza vaccination in immunodeficient patients, and discusses available information on the effect of immunoglobulin products on seasonal influenza susceptibility and severity in PAD patients receiving IgRT. PubMed was searched up to 20 November 2023 using the search parameters “influenza and antibody immune deficiency”, “influenza and immunoglobulin replacement therapy” and influenza vaccination and immunodeficiency”. Priority was given to articles published in the past 5 years and the bibliography was augmented with papers known to the authors or identified in the discussion sections of the selected references. The search was limited to articles published in the English language.

## Seasonal influenza

Seasonal influenza tends to peak during the winter months in temperate regions, whereas the seasonal pattern is less distinct in tropical regions, where influenza occurs at various times of year or all year-round (13). The exception to this epidemiological behavior occurred in the 2021-2022 and 2022-2023 post-pandemic seasons when two different peaks more than 4 weeks apart took place in many European and American countries (1, 14).

The influenza virus is mainly transmitted via respiratory droplets and has an incubation period of 18–72 hours (15, 16). Influenza usually manifests as an acute febrile illness, with symptoms including fever, chills, dry cough, headache, sore throat, rhinitis, myalgia and malaise (16). Most patients will have a self-limiting illness, with full recovery. However, complications can develop in some patients, the most significant of which is pneumonia. Acute otitis is more common in children, but myositis, sinusitis, other ear infections, neurological and cardiovascular disorders can also develop in all age groups, particularly in high-risk patients (16).

**Figure 1:**
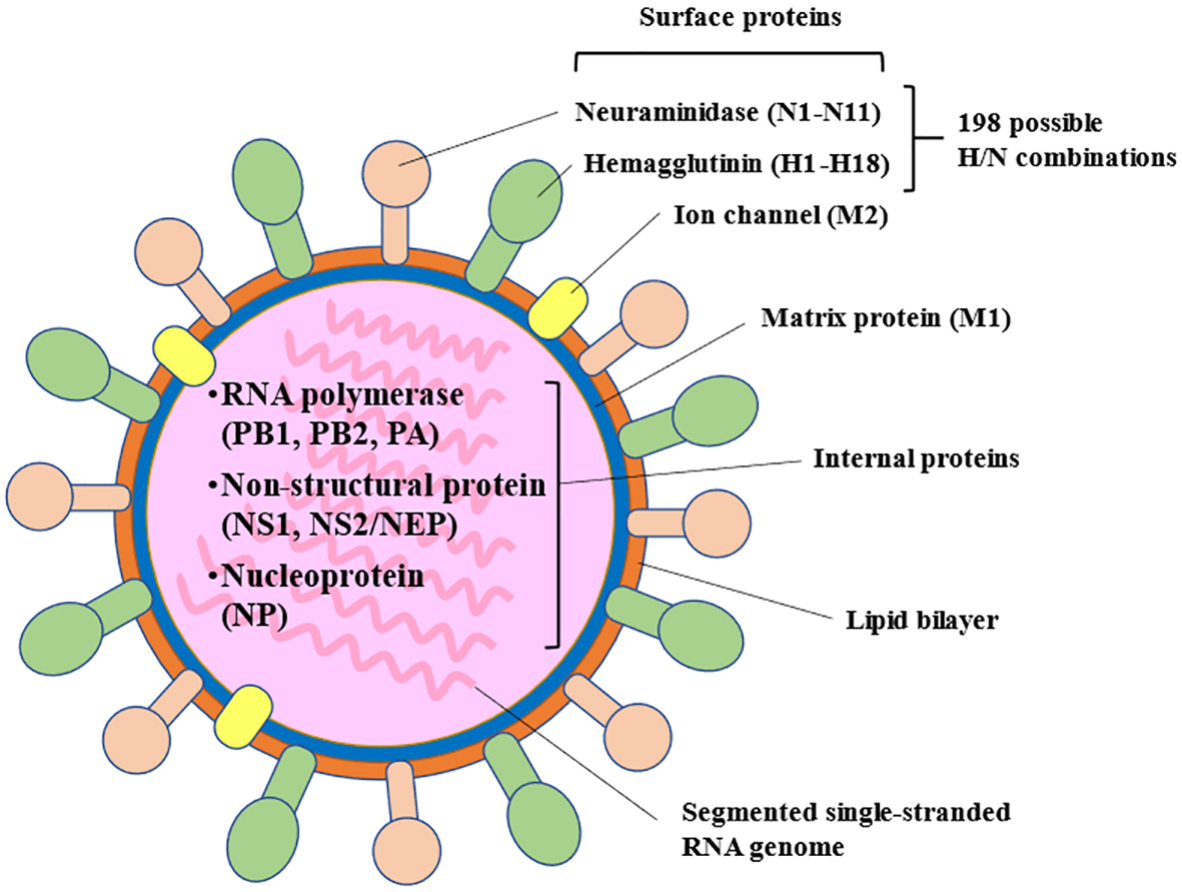
Influenza A virus structure: internal and surface proteins.

Among the types of influenza virus that infect humans, influenza A is the most common cause of seasonal influenza infection, followed by the influenza B virus. The influenza C virus never causes epidemics, only mild illness in individual cases or small outbreaks (16, 17). Influenza A viruses are classified based on their surface glycoproteins, including hemagglutinin, which is involved in binding to host cell surfaces enabling entry into the cell. Neuraminidase is involved, importantly, in the release of new virions from the host cell and its diffusion through the organism, and is considered to be a virulence factor (15) (**Figure 1**). Influenza viruses, especially influenza A virus, undergo frequent mutations, which can cause antigenic changes to hemagglutinin and, to a significantly lesser frequency, neuraminidase. Accumulation of these mutations can lead to slightly different strains circulating each year (antigenic drift) (15, 16). In addition, reassortment of genetic material from two influenza strains of different subtype can lead to the emergence of new subtypes (antigenic shift) with pandemic potential (15, 16).

Seasonal influenza vaccines are formulated each year at different dates for each hemisphere, based on the strains that are predicted to be circulating that season (15). Trivalent vaccines generally contain two strains of influenza A and one lineage of influenza B (Victoria lineage); quadrivalent vaccines contain an additional B lineage (Yamagata lineage) (16). Before 2023 it was recommended to include two strains of type B (one from each of the Victoria and Yamagata lineages), but after confirming the disappearance of Yamagata lineage in 2023, the WHO strain selection committee for influenza vaccines no longer recommend quadrivalent vaccines (18).

Studies have shown that the immune response for influenza strains encountered earlier in life is biased in future encounters to antigenically related strains of influenza (19). This immune process, first recognized in 1960, is called immune imprinting where the first exposure to viral antigens shapes the immune response to subsequent infections with the same virus, e.g. influenza (20). However, this back-boosting of antibodies to preceding influenza strains may lead to a reduction in the response to new viral strain epitopes (21). Nevertheless, previous exposure history to influenza (and other viruses) may contribute to protection from influenza strains within the same phylogenic group and may also be important in vaccine responses. The effectiveness of the vaccine depends on the immune imprinting to the first flu encounter, the age and health of recipients, the type of vaccine, and how well the vaccine strains match the strains in circulation (3). The Center for Disease Control and Prevention (CDC) has estimated the influenza vaccine effectiveness from 2004 to 2023 to range from a low of 10% in 2004-2005 to 60% in 2010-2011; the estimate for 2022-2023 is 54% (22) with differences among children and adults in the same season.

## Burden of influenza disease in immunodeficient patients

Patients who are immunocompromised generally experience more severe influenza illness and more complications than non-immunocompromised patients (23, 24). This is particularly evident in adults with SID, such as that associated with malignancy, immunosuppressive drugs, and hematopoietic stem-cell transplantation (23–26).

Significant influenza-associated morbidity has also been reported for pediatric oncology patients (27). However, studies that included a broader range of immunocompromised children (most of whom had SID) found that, although they were more likely to be hospitalized or to have a longer hospitalization than non-immunocompromised children, they were less likely to require intensive care or to die (28, 29). One possible explanation for the latter is that immunocompromised children might be admitted with less severe illness than other children, in order to monitor them closely because of their underlying immunocompromised status (28).

Influenza infection is a cause of exacerbations in patients with chronic pulmonary diseases. Huang et al. reported that patients with bronchiectasis who contracted influenza illness had a high risk of respiratory failure and death (29). Similarly, since PAD patients develop chronic lung disease such as bronchiectasis they may also be at risk of serious and potentially life-threatening illness from influenza infection (30–33). There is very limited information about the prevalence of influenza infection in this population. A self-reported survey study by Ballow et al. involving more than 1000 patients with PAD in the USA noted that, despite a high level of exposure to the virus (74% in children, 46% in adults), only 17% of patients were diagnosed with influenza (31). A small study by Ponsford et al. involving 41 patients with PAD and symptomatic respiratory exacerbation who underwent nasal swab testing found that 13/870 (1.5%) swabs were positive for influenza virus, which was similar to a control group (1.4%) (32). Sperlich et al. reported that influenza virus was detected in 2 of 54 patients (3.7%) with common variable immunodeficiency (CVID) and symptomatic respiratory exacerbations who underwent nasopharyngeal swab testing (33).

Although patients with PAD are generally at increased risk of severe infections, patients who develop influenza do not necessarily become severely ill. Nanishi et al. reported that among 910 PID patients, only 20 (2.2%) were hospitalized with influenza, and among 18 patients for whom relevant data were available, only one required mechanical ventilation and none died (34). The incidence of hospitalization for severe complications of influenza in Japanese patients under age 75 between 2012-2016 was 1.0% [mainly pneumonia, acute respiratory failure, febrile seizures, encephalitis/encephalopathy, and acute respiratory distress syndrome] (35). There are several factors that could help explain why influenza in PAD patients is not more severe or occur more frequently than in immunocompetent individuals, and these include: greater adherence to general prophylactic measures, such as good hygiene (e.g. hand washing) and social distancing (12); treatment with IgRT (10, 11); receipt of prompt antiviral therapy after symptom onset (34); and they, and their close family contacts, recognize the importance of seasonal influenza vaccination (10, 12, 36). In a study by Cox et al. in Ireland, PIDD patients receiving IgRT were surveyed about seasonal influenza vaccine for patients and family members (37). A total of 76% of IgRT patients received the influenza vaccine. However, many were not aware that vaccination was recommended for household members, and only 24% had full vaccine coverage at home. Similarly, a study by Ballow et al. reported that 75% of patients had received the influenza vaccine (31).

## Can immunodeficient patients mount an immune response to influenza vaccine?

The adaptive immune response to influenza infection involves antibody and cellular responses (38, 39). Neutralizing antibodies to the surface glycoproteins hemagglutinin and neuraminidase are central to protection against influenza infection and disease (38, 39). The importance of a T-cell immune response is less clear, but studies indicate that it is important for influenza virus clearance and the recovery from influenza viral infections, and may be relevant for reducing the severity of the infection and complications (40–43). Studies in mice found that T-cells can increase protection from influenza virus infection through mobilization of immune effectors, and this was independent of B-cells and immunoglobulin (44–46).

In patients with SID, the serological response to seasonal influenza vaccination tends to be weaker than seen in immunocompetent controls, but vaccination still reduces the risk of influenza-like illness and severe disease (9, 47). A meta-analysis by etiology of immunocompromise found that vaccination was associated with significantly lower odds of influenza-like illness in patients with HIV or cancer, and transplant recipients compared with placebo or no vaccination. However, the odds of seroconversion and seroprotection were generally lower in these patient groups compared with immunocompetent controls (48).

Immunization against seasonal influenza is recommended for patients with PAD (10–12, 49). However, the humoral immune response to seasonal influenza vaccination is reduced in this patient population (generally patients with CVID), although protective antibody levels were achieved in some patients (43, 50, 51). Data regarding cellular immune response to the influenza vaccine were generally favorable, but they were not always consistent; see discussion below (43, 51). None of the studies evaluating immune responses reported clinical outcomes after influenza vaccination.

Van Assen et al. noted that patients with humoral immunodeficiency (n=26 including 18 with CVID) did not develop protective anti-influenza antibody titers after vaccination with a trivalent vaccine (52). Kostinova et al. found that protective antibody levels were not achieved in most patients in a small group of CVID patients (n=6) given a quadrivalent vaccine (53). A study by Zhan et al. showed that CVID patients generally had a poor humoral response to a trivalent influenza vaccine, and that half of the patients were not able to generate influenza-specific memory B cell responses (54).

Hanitsch et al. studied both humoral and cellular immune responses to influenza following trivalent vaccination in a small group of CVID and ‘unclassified antibody deficiency’ patients, and found that while only 1 of 8 CVID patients and 4 of 8 ‘unclassified antibody deficiency’ patients mounted a specific antibody response to influenza vaccination, most of the CVID (7/8) and ‘unclassified antibody deficiency’ (6/8) patients mounted a cellular immune response as measured by vaccine-induced T cells (the frequency of CD40L+ T cells producing interferon-γ, tumor necrosis factor-α, and interleukin-2) (55). Similarly, Friedmann et al. studied the immune response to a quadrivalent influenza vaccine in patients with CVID (n=17) and found that only one patient mounted a humoral immune response to vaccination, but most patients showed an increase of T follicular helper cells and an increase in antigen specific CD25^+^OX40^+^ T cells (56). In contrast, evaluation of cell-mediated immune response in a subgroup of patients with CVID from the study by van Assen et al. (n=15) found no increase in CD4+ T cells producing IFN-γ after influenza vaccination (57).

Despite the poor humoral response, it is currently recommended that PAD patients should be given inactivated influenza vaccine because of the reported cellular response and the speculated lack of protective antibody levels against the current influenza strain in the immunoglobulin preparations used for replacement therapy (10, 49). However, as noted later in the review, cross-reactive antibodies to current seasonal influenza strains have been documented (see “*Immunoglobulin products contain antibodies to influenza”* section below) and some level of protection against influenza infection may be anticipated.

## Immunoglobulin replacement therapy

IgRT is administered to patients with significant antibody immune deficiency with the main goal of providing passive immunity against infections and reducing the risk of complications (58). Consequently, PAD patients generally receive IgRT (10, 58–61). It is also recommended for patients with secondary hypogammaglobulinemia if the patient is experiencing recurrent or severe infections and management with prophylactic antibiotics has not been successful (62).

Intravenous (IVIG) and subcutaneous (SCIG) immunoglobulin therapies contain polyclonal, polyspecific immunoglobulins, predominantly IgG, obtained from pooled plasma donations from several thousand individuals (63, 64). Donors will have been naturally exposed to many potentially pathogenic micro-organisms and will have received various vaccinations, and therefore their plasma contains a range of pathogen-specific antibodies (64, 65). Given that IgRT products use pooled plasma from a large number of donors, the range of antibody specificities present in the products will be much broader than would be seen in plasma from any single individual, but specific antibodies found in a few plasma donor individuals will not contribute much to the final plasma pool.

### Immunoglobulin products contain antibodies to influenza

It is known that IgRT products contain neutralizing antibodies against influenza virus (**Table 1**) (66–68). However, the production of IgRT lots generally takes 9 months to complete, and donor plasma may be collected several years before the manufacture of IgRT products (69). Therefore, given the frequent changes in seasonal influenza virus strains due to antigenic drift, it is generally assumed that IgRT products contain antibodies against virus strains that were circulating prior to the time of donation (i.e. in the past), and it is believed that immunoglobulin products will not necessarily have sufficient coverage against currently circulating seasonal influenza strains. The potential for IgRT to protect against influenza infection depends on the range of antibodies present in the product, and any cross-reactivity between antibodies to different strains of influenza in the population, especially those directed against HA, but also the more conserved surface antigen of neuraminidase, and M protein.

**Table 1:** Antibodies to influenza virus in immunoglobulin replacement therapy products.

Study	IgRT products	Tests	Results
Yunoki et al., 2010 (66)	IVIG lots (Kenketsu Venoglobulin^®^-IH) manufactured in 1999 (2 lots) and in 2008 (3 lots)	HI, VN against pandemic H1N1 (2009), seasonal H1N1 (2008), vaccine H1N1 (1999), classical swine H1NI (1981) influenza A	1999 IVIGs: HI and VN titers of 4–16 and 32–64 respectively against pandemic (2009) and classical swine (1981) H1N1, and 4–40 and 8–128 respectively against seasonal (2008) and vaccine (1999) H1N1. 2008 IVIGs: HI and VN titers of 8 and 64 respectively against pandemic (2009) and classical swine (1981) H1N1, and 20–320 and 160–1280 respectively against seasonal (2008) and vaccine (1999) H1N1. Conclusion: Both the 1999 and 2008 IVIGs showed activity against pandemic (2009), classical swine (1981), seasonal (2008) and vaccine (1999) H1N1, indicating IVIG had cross-reactivity against different H1N1 strains.
Hong et al., 2011 (67)	IVIG products manufactured before 2009 H1N1 influenza A pandemic, including Gamunex (3 lots); Cytogam; Gammagard; Privigen	HI, MN against 2009 pandemic H1N1	All IVIG products demonstrated dose-dependent increases in HI titers. All IVIG products demonstrated dose-dependent increases in MN titers, and achieved a MN titer of 1:20 at a concentration of 2.0 g/dL. Conclusion: Prepandemic IVIG products contained significant levels of cross-reactive specific antibody against 2009 H1N1.
Kubota-Koketsu et al., 2012 (68)	IVIG lots manufacture in Japan and USA between 1993 and 2010	HI, VN against H2N2 (‘Asian flu’ prevalent 1957–1967)	All IVIG lots contained high HI and VN activity against H2N2. Both HI and VN titers were higher against isolates from 1965 (HI: 64–128; VN_50_: 1280–5120) than isolates from 1957 (HI: 32–64; VN_50_: 80–320). Conclusion: Anti-H2N2 antibody titers in IVIG products correlated with history of infection and vaccination programs.
Díez et al., 2022 (71)	IVIG products manufactured in June 2020 (from plasma collected in prior 6 months), including Flebogamma DIF and Gamunex	HI, VN against WHO-recommended influenza vaccine strains for N. hemisphere 2020–2021 and 2021–2022 and S. hemisphere 2021 seasons	The IVIG products had high HI titers (1:320–1:3200) and high infectivity neutralization activity (VN_50_ 1:2399–1:21,428) against all influenza strains tested. Conclusion: IgRT products produced during a single influenza season showed potent activity against influenza strains included in worldwide vaccines during three consecutive influenza seasons.
Onodera et al., 2017 (69)	IVIG products manufactured 1999-2014, including Venoglobulin (1999–2014), Nisseki Polyglobin-N 5% and 10% (2013-2014)	HI, VN against seasonal influenza H1N1, H3N2 and influenza B vaccine strains	All IVIG lots had HI and VN activity against all seasonal influenza strains. Activities were stable over short periods of time, but increased slowly over extended periods. Conclusion: Donor populations and IgRT products maintained specific and cross-reactive antibodies against seasonal influenza over extended periods of time.
Jegaskanda et al., 2014 (78)	Eight IVIG (Sandoglobulin) lots manufactured before 2009 H1N1 pandemic and 10 manufactured after 2010	Detection of ADCC-mediating antibodies against 2009 pandemic H1N1, avian influenza H5N1 and H7N9	ADCC-mediating antibodies to H1N1 hemagglutinin detected in all IVIG products. ADCC-mediating antibodies to H1N1 neuraminidase were detected in some prepandemic and all postpandemic IVIG. Hemagglutinin-specific ADCC were able to recognize a broad range of hemagglutinin proteins including those in H5N1 and H7N9. Conclusion: IVIG products contained broadly cross-reactive ADCC-mediating antibodies.

ADCC, antibody-dependent cellular cytotoxicity; HI, hemagglutination inhibition assay; IgRT, immunoglobulin replacement therapy; IVIG, intravenous immunoglobulin; MN, microneutralization assay; VN, virus neutralizing assay; VN_50_, Dilution producing 50% neutralization.

However, there is evidence that, even with antigenic drift and antigenic shifts, specific antibodies and cross-reactive antibodies to the current seasonal influenza virus are present in available immunoglobulin products (**Table 1**) (70). Díez et al. tested IVIG that was manufactured in June 2020 (using plasma collected in the previous 6 months) against influenza strains that were included in vaccines for the period 2020–2022 (Northern hemisphere 2020–2021 and 2021–2022 seasons and Southern hemisphere 2021 season) and found high hemagglutination inhibition (HI) titers and viral neutralization (VN) titers against all of the strains (71). This highlights that in addition to containing neutralizing antibodies against hemagglutinin (detected by the HI assay), the IVIG product also contained neutralizing antibodies against other influenza surface proteins such as neuraminidase (indicated by the VN assay, which detects neutralizing antibodies against various surface proteins, including hemagglutinin and neuraminidase). Onodera et al. measured HI and VN titers to A/H1N1, A/H3N2 and B influenza vaccine strains in various lots of IVIG manufactured in different years between 2009 and 2014, and found that IVIG products contained significant HI and VN titers against all studied strains (69) and possibly against other internal important proteins. They concluded that that the donor population has both specific and cross-reactive antibodies against seasonal influenza viruses, even given yearly changes in influenza viral antigenicity.

This would be consistent with data from a study performed after the 2009 H1N1 influenza A pandemic, which found that 34% of serum samples from individuals older than 60 years contained cross-reactive antibodies to the pandemic influenza virus, which was thought to be due to priming from natural infection with H1N1 previous influenza virus strains (72). Furthermore, Yunoki et al. found that immunoglobulin products produced in 1999 and 2008, before the H1N1 pandemic in 2009, contained HI and VN titers against 2009 pandemic H1N1, as well as classical swine H1N1 and seasonal H1N1 influenza viruses (66).

Preclinical studies have demonstrated that the specific and cross-reactive antibodies in IgRT products are protective (**Table 2**). Hohenadl et al. found that IVIG containing high titer antibodies to the 2009 pandemic H1N1 virus provided complete protection from mortality due to challenge with the virus in immunodeficient SCID mice (73). There was a dose-dependent correlation between levels of circulating hemagglutinin and neutralizing antibodies measured on the day of challenge and survival. The study also demonstrated there was enrichment of both hemagglutinin- and neuraminidase-specific antibodies in the IVIG preparation. Rockman et al. showed that IVIG prepared before the 2009 H1N1 pandemic prevented viral replication in the lungs of ferrets challenged with the pandemic strain (74). It also provided dose-dependent protection against morbidity and mortality in animals exposed to an otherwise lethal challenge with highly pathogenic avian H5N1 influenza.

**Table 2:** Preclinical studies evaluating protective effect of anti-influenza antibodies in immunoglobulin replacement therapy products.

Study/Model	IgRT product or IgG fragment	Tests	Results
Hohenadl et al., 2014 SCID mouse challenge (73)	IVIG manufactured before 2009 H1N1 influenza A pandemic and post-pandemic hyperimmune IVIG	HI, MN, Nai, and protection against pandemic H1N1 and seasonal H1N1	Prepandemic IVIG contained substantial levels of HI, MN and Nai antibodies against pandemic H1N1 (1:45, 1:204, 1:727, respectively) and seasonal H1N1 (1:688, 1:4946, 1:312, respectively). Titers were 28-, 56- and 3.4-fold higher in postpandemic hyperimmune IVIG. Postpandemic hyperimmune IVIG provided protection from death for SCID mice challenged with pandemic H1N1 (100% survival at 29 days), whereas prepandemic IVIG did not (50% survival at 29 days versus 40% in buffer control group). Conclusion: Hyperimmune IVIG could potentially provide prophylaxis against pandemic influenza in high-risk groups.
Rockman et al., 2017 Healthy juvenile ferret model (74)	IVIG manufacured before 2009 H1N1 pandemic, including Intragram^®^P, Privigen^®^, Hizentra^®^	HI, VN, and protection against pandemic H1N1 and highly pathogenic avian influenza H5N1	IVIG prevented significant viral replication in the lung (but not the upper respiratory tract) after pandemic H1N1 challenge. IVIG significantly reduced morbidity and mortality after exposure to an otherwise lethal challenge with H5N1 versus controls given buffer (survival p=0.0058). Survival was significantly better in animals given IVIG F(ab)’2 (80%) versus Fc-treated (17%) and diluent control (10%) groups (p=0.0015). Conclusion: Prepandemic IVIG modulated influenza-associated mortality. A specific-cross-reactive antibody was likely responsible.
Lu et al., 2006 BALB/c mouse model (75)	Equine anti-H5N1 hyperimmune IgG F(ab)’2 fragments	HI, VN, and protection against H5N1 infection (survival rate assay)	H5N1-specific F(ab)’2 fragments had HI titer 1:1024 and VN titer 1:2048. F(ab)’2 fragments protected mice infected with an otherwise lethal dose of H5N1; doses of 100 and 200 μg provided 100% protection (versus 0 survival in the antibody-negative control group). Conclusion: H5N1-specific F(ab)’2 fragments provided protection from mortality due to H5N1 infection.
Ramisse et al., 1998 BALB/c mouse model (76)	IVIG and F(ab)’2 fragments	HI, and protection against H3N2 infection	HI against H3N2 was 1/16. IVIG administered 3, 6 or 24 hours before H3N2 challenge provided dose-dependent protection against influenza pneumonia (survival at 24 hours: 12/20 animals at 62.5 μg, 16/20 at 125 μg, and 19/20 at 250 μg). Intranasal F(ab)’2 fragments administered 24 hours pre-challenge provided protection at 150 μg (survival 10/10) but not at 100 μg (2/10). In the control group, survival was 2/30 at 24 hours. Conclusion: IVIG and topical F(ab)’2 provided prophylaxis against H3N2 pneumonia.

HI, hemagglutination inhibition assay; IgRT, immunoglobulin replacement therapy; IVIG, intravenous immunoglobulin; MN, microneutralization assay; Nai, neuraminidase inhibition; VN, virus neutralizing assay; VN_50_, Dilution producing 50% neutralization.

Both anti-hemagglutinin and anti-neuraminidase antibodies are found in polyclonal immunoglobulin preparations (73, 74). Preclinical studies have found that the F(ab’)2 fragment of IVIG, but not the Fc fragment, protects against influenza virus infection in ferrets (74) and mice (75, 76). This suggests a specific antibody-mediated mechanism of protection, and that complement or Fcγ-bearing cells were not necessary for protection in these particular models (74, 76, 77). Meanwhile, a study that evaluated a range of IVIG products found that they contained broadly cross-reactive influenza-specific antibodies that mediated antibody-dependent cellular toxicity *in vitro* (78).

Overall, these studies suggest that significant levels of specific and cross-reactive antibodies are present in pooled plasma used to prepare IgRT products, which could potentially serve to protect antibody deficient patients.

Similar observations have been published regarding the presence of antibodies in IVIG products to severe acute respiratory syndrome coronavirus-2 (SARS-CoV-2), associated with coronavirus disease-2019. Cross-reactive neutralization between SARS-CoV-2 and SARS-CoV, but not Middle East respiratory coronavirus (MERS-CoV) syndrome, has been reported for some IVIG products that were manufactured before the COVID-19 pandemic (79). Studies since 2021–2022 indicate that most commercial IVIG products contain neutralizing anti-SARS-CoV-2 spike antibodies, although there is some variation in the level between products (80–83). A recent study found that when this IVIG was administered to antibody-deficient patients, an increase in anti-spike antibody titers was generally seen in their serum (80). Romero et al. reported that IVIG products contained anti-SARS-CoV-2 antibodies with neutralizing activity against different variants of the virus, including wild-type virus, and the alpha, beta, gamma and delta variants of concern, which reinforces the concept of cross-reactivity across variants (83).

## Are the antibodies to influenza in immunoglobulin products protective against seasonal influenza?

Although there is evidence from preclinical and *in vitro* studies that IgRT may contribute to prophylaxis against influenza, there are very few clinical data.

Ballow et al. undertook a survey of PAD patients enrolled in the database of the Immune Deficiency Foundation during the 2016–2017 influenza season (31). The analysis included data from 1009 individuals aged 2 to 88-years-old who had X-linked agammaglobulinemia (n=41; XLA), CVID (n=824) or hypogammaglobulinemia (n=144). Although the rate of contact with other people with influenza was high (74% in children and 46% in adults), most patients (83%) did not report an influenza-like illness.

**Figure 2:**
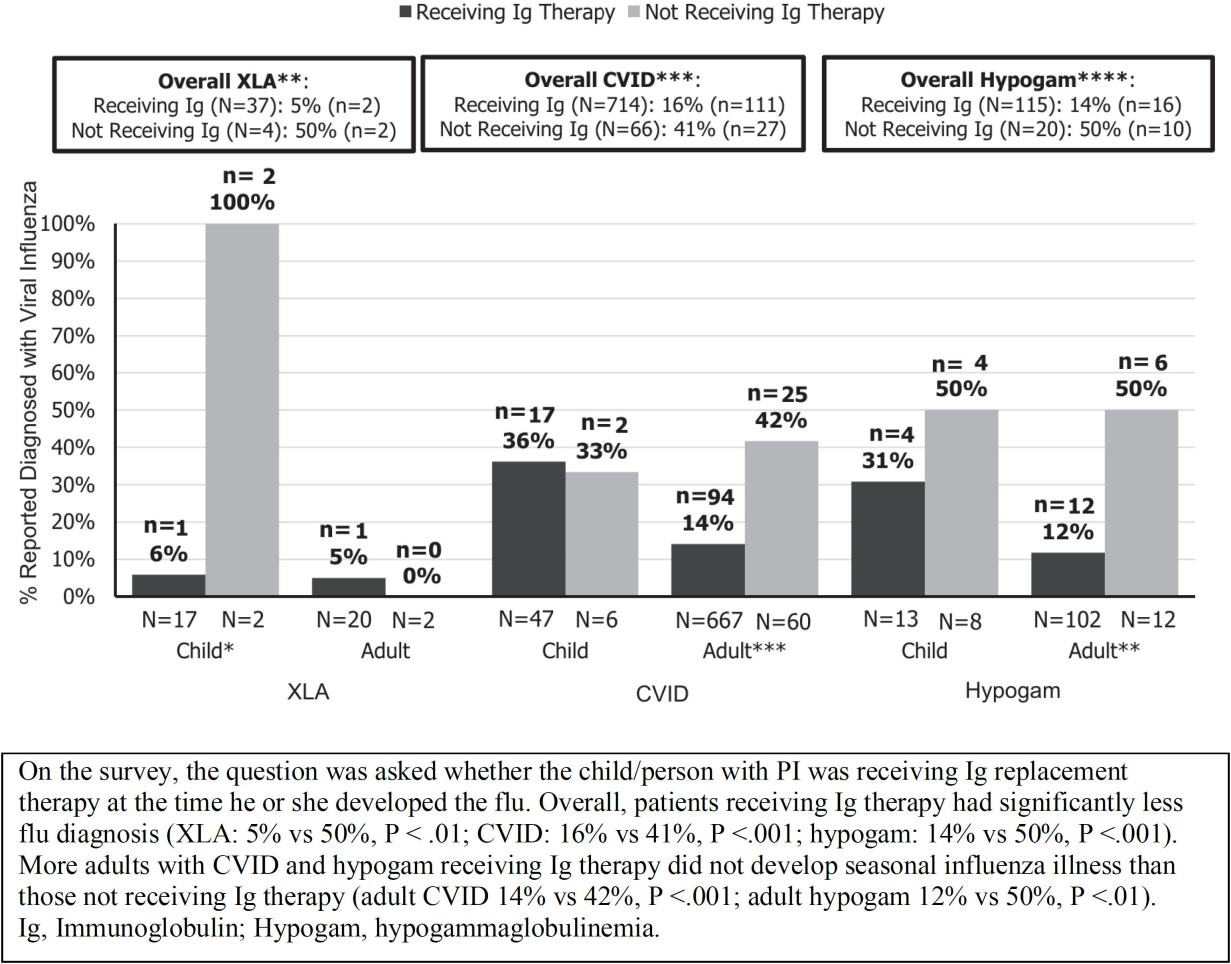
Diagnosis of seasonal influenza in patients with primary antibody immunodeficiency according to whether or not patients were receiving immunoglobulin replacement therapy (reproduced with permission from 31). *p<0.05, **p<0.01, ***p<0.005, ****p<0.001.

XLA patients cannot produce specific antibodies in response to influenza vaccination, but only 4 of 41 participants (10%) with XLA were diagnosed with influenza who were receiving IgRT. Among children with XLA, only 1 of 17 who were receiving IgRT was diagnosed with influenza, whereas both children who were not on IgRT were lab-confirmed for influenza. Similarly, among adults with XLA, only 1 of 20 patients on IgRT was lab-confirmed for influenza. Consistent results were seen in CVID patients, with only 14% of adults on IgRT therapy developing influenza compared with 42% of those not on IgRT (p<0.001) (31) (**Figure 2**).

Overall, 75% of patients had received a seasonal influenza vaccination, and so it is possible that patients may have produced some protective antibodies and/or cellular immunity in response to the vaccine. In addition, as the study was based on a patient self-reported survey, influenza illness was not confirmed based on physician information or laboratory flu tests. Nevertheless, only 17% of those surveyed were diagnosed with influenza despite high rates of exposure to influenza illness. The authors concluded that, overall, the results suggested that IgRT may have modified the risk of seasonal influenza in PAD patients (31). Gokturk et al. reported observations on immunocompromised children hospitalized with H1N1 influenza during the pandemic; 62% were under 5 years and 58% had a PID. None of the 6 PID patients who received regular IVIG replacement therapy needed intensive care or died (84).

## Discussion

To provide adequate protection against infectious diseases, IgRT is standard therapy for patients with PAD since it significantly reduces overall risk for pneumonia (85). However, PAD patients who are receiving IgRT continue to have a substantial burden of viral and bacterial pathogens in the upper respiratory tract (32). The effectiveness of therapy is clear for some common infectious pathogens, particularly bacteria, including those responsible for pneumonia (60, 62, 86). Nevertheless, there is less data for other infectious agents, such as viruses. Protection against influenza is a particular case, as it is widely accepted that IgRT products cannot protect against future influenza virus infections due to antigenic drift. Nevertheless, as discussed in this paper, there is evidence that significant levels of specific and cross-reactive antibodies to influenza virus strains are present in IgRT products and this suggests that IgRT could contribute to protection against seasonal influenza in PAD patients. However, there is currently a limited amount of clinical evidence to support this (31, 84, 87). The best available findings come from a retrospective survey of PAD patients, for which it was not possible to obtain physician or laboratory confirmation of influenza diagnoses (31). Nonetheless, the study found that the rate of influenza was lower among patients who were receiving IgRT compared to those PAD patients not receiving IgRT, despite a high level of exposure to other people with influenza. Larger, prospective clinical studies are needed to confirm whether regular IgRT has a significant effect on the risk or severity of seasonal influenza infections in patients with PAD.

We have focused on seasonal influenza in this paper. However, IgRT potentially also has a role in protecting against pandemic influenza. IgRT products contain broadly cross-reactive antibodies (66, 78), and animal studies have shown that pre-pandemic IgRT products can provide protection against challenge with pandemic influenza strains (73, 74). In the small series reported by Gokturk et al., PID patients on regular IgRT who became infected with the H1N1 pandemic virus had a less severe clinical presentation (84). In addition to the protective role of anti-hemagglutinin antibodies, cross-reactive antibodies to neuraminidase may also play a protective role during pandemics. Thus, during an influenza pandemic, IgRT could possibly provide a degree of protection for high-risk individuals if the new reassortant virus has common or related main surface antigens.

## Conclusion

There is laboratory and anecdotal evidence that IgRT could contribute to protection against seasonal influenza in antibody deficient patients. Now, additional clinical data are needed to confirm the extent and relevance of this protection.
